# Supplementary material for: Spinal Cord Stimulation Improves Deceleration Phase Control during Targeted Reaching Post-Stroke
Source: Res Sq. 2026 Feb 18:rs.3.rs-8752608. Preprint. [Version 1] doi: 10.21203/rs.3.rs-8752608/v1 (PMC12934899; doi:10.21203/rs.3.rs-8752608/v1)
Supplement: 1 [file NIHPPRS8752608V1-supplement-1.pdf]

Supplementary information

Interaction Torque Contributions and Choice of Control Gain Structure

For single joint movements, torque is related to angular acceleration simply by a single factor (i.e. moment of inertia of the arm segment moving). In two-joint arm movements, movement of one joint can generate torque at the other (i.e. interaction torques). Interaction torques are typically more prominent at some arm configurations over others (e.g. fully straight arm versus elbow and shoulder slightly flexed), and at higher angular speeds of either joint, as can be inferred from the equations of motion. In all the planar movement trials we performed, due to relatively lower speeds and ranges of arm configurations, torques of the elbow and the shoulder were mostly dominated by torque generated at individual joints. That is, interaction torques were quite minimal, especially for stroke participants due to their lower reaching speeds. This justifies the choice of control model where each joint is controlled independently from the other (i.e. the elbow PD controller only generates torque based on elbow feedback, and same for the shoulder). Plots of example interaction and generated torques for shoulder and elbow joints of one healthy and two stroke participants are shown below to illustrate the idea.

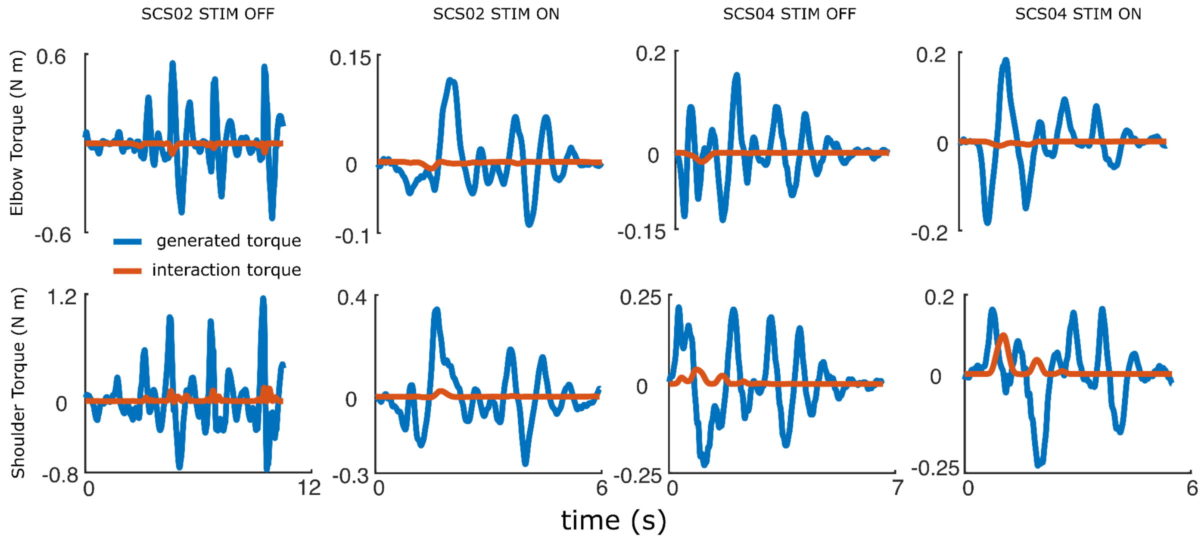

Supplementary Figure 1. Interaction torques were often not very prominent in the task we were modeling, likely due to small angle ranges and relatively slow reaching speeds.

Non-paretic arm versus healthy model fits

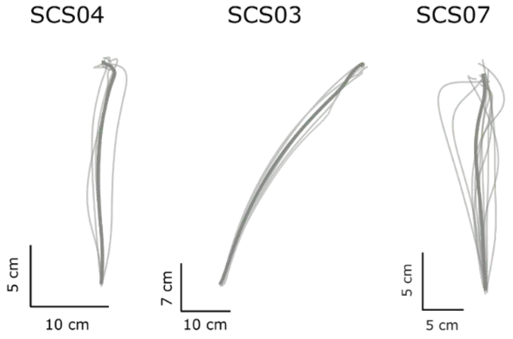

Supplementary Figure 2. Non-paretic arm showed closer to healthy point-to-point reaching. Non-paretic arm reaching was not

recorded during planar arm reaching for SCS02.

Supplementary Videos

Supplementary Video 1: HC09 component torques

Supplementary Video 2: SCS02 STIM OFF component torques

Supplementary Video 3: SCS02 STIM ON component torques

Supplementary Video 4: Simulated arm with control structure tuned to produce smooth reaching.

Supplementary Video 5: Simulated arm with suboptimal control structure (increased flexor derivative gain).
